# Complete Genome Sequences of *emm*6 *Streptococcus pyogenes* JRS4 and Parental Strain D471

**DOI:** 10.1128/genomeA.00725-15

**Published:** 2015-07-02

**Authors:** Gary C. Port, Elyse Paluscio, Michael G. Caparon

**Affiliations:** Department of Molecular Microbiology, Washington University School of Medicine, St. Louis, Missouri, USA

## Abstract

We report the complete genome assemblies of the group A *Streptococcus pyogenes* serotype *emm*6 strain D471 and its streptomycin-resistant derivative JRS4. Both of these well-studied laboratory strains have been extensively characterized over the past three decades and have been instrumental in the discovery of multiple aspects of streptococcal pathogenesis.

## GENOME ANNOUNCEMENT

*Streptococcus pyogenes* is a Gram-positive bacterial pathogen responsible for a broad range of human diseases (1). The genome of *S. pyogenes* encodes an arsenal of adhesins and toxins that enable this strict human pathogen to infect a wide range of human tissues. Immunity toward *S. pyogenes* is strain specific, as each strain encodes a unique set of surface antigens known as M-protein (2) and T-antigen (3). Advances in streptococcal genetics over the past several decades have facilitated the detailed characterization of numerous virulence factors. Much of the pioneering work in this field has utilized a strain from the Rockefeller University Lancefield collection known as D471, a rheumatic fever-associated M6 isolate, as well as its streptomycin-resistant derivative, JRS4 (4). These studies include the first targeted gene deletion (5), chromosomal complementation (6), and isogenic replacement of different M-protein encoding genes (7). Additionally, the M-protein regulator Mga was first identified (8) and episomally complemented (9) in these strains. Furthermore, the alternative sortases that covalently link T-antigen (pilus) to the cell wall were discovered in these strains (10, 11). Finally, these strains were used to first describe cytolysin-mediated translocation (CMT), whereby the secreted effector SPN is translocated into host cells by the pore-forming cytolysin SLO (12). As these strains were and continue to be heavily investigated, we sought to determine the complete genome sequences of JRS4 and D471 in order to provide a framework for future genetic studies on these classic strains.

Genomic DNA (gDNA) from JRS4 was purified by phenol chloroform extraction (13), and sequenced using a 454-GS FLX sequencer (MOgene LC, St. Louis, MO) by collecting shotgun reads and 8-kb paired-end reads as previously described (14). A total of 211,893 reads (67,091,661 nucleotides) were generated, reaching 37-fold genome coverage depth. Sequences were assembled into 26 contigs using Newbler v2.5.3, and were aligned to the SF370 *S. pyogenes* genome (15), generating a single scaffold that was 97% complete. The remaining gaps (ranging from 0.3 kb to 15 kb, total of 58 kb) were filled in by primer walking (IDT, Coralville, IA) and Sanger sequencing (GENEWIZ, South Plainfield, NJ). To correct sequencing errors, gDNA was resequenced by Illumina HiSeq 2000 (GTAC, Washington University, St. Louis, MO) by collecting 50-bp single-end reads generating a total of 7,763,695 reads (301,814,052 nucleotides) reaching 167-fold genome coverage depth. Illumina data were aligned to the reference JRS4 scaffold sequence using DNASTAR SeqMan NGen 4.0.0 (DNASTAR) to generate a final consensus sequence. gDNA from D471 was purified and sequenced by Illumina HiSeq2000 generating a total of 4,359,256 reads (214,875,135 nucleotides) reaching 119-fold genome coverage depth, and aligned to the reference JRS4 scaffold sequence as described above. The JRS4 and D471 genomes are composed of 1,811,968 bp, with an average G+C content of 38.6%. JRS4 contains 6 single-nucleotide polymorphisms (SNPs) compared to its parent D471 including a nonsynomymous substitution in *rpsL* (N56K), which confers streptomycin resistance. The remaining SNPs are in *cypB* (S233T), *rplS* (S40I), *fabT* (F35L, T51I) (16, 17), and an SNP in a noncoding intergenic region 175 bp upstream of *prfC*.

### Nucleotide sequence accession numbers.

The complete whole-genome sequences of *S. pyogenes* strains JRS4 and D471 have been deposited at NCBI GenBank under the accession numbers CP011414 and CP011415 with locus tags SpyM6JRS4 and SpyM6D471, respectively.

## ADDENDUM IN PROOF

During the preparation of this manuscript, a JRS4 genome sequence was deposited in GenBank by the Tokyo Medical and Dental University with the locus tag SPYJRS4 under the accession no. AP012335.1. Remarkably, comparison between the two JRS4 genomes revealed only 44 regions of difference, including 39 indels and 5 SNPs throughout the > 1.8 million nucleotides. The majority of differences (37 indels) reside in homopolymeric stretches of adenines/thymidines (between 4- and 9-nucleotide-long stretches), 23 of which occur in intergenic regions, while the remaining reside in open reading frames (ORFs) and would primarily result in frameshift mutations and early stop codons. Comparison of these ORFs with the 20 *S. pyogenes* genomes available in the KEGG database reveals these mutations to be unique to the SPYJRS4 genome sequence. Although homopolymeric nucleotide stretches are subject to slip-strand mutation (16), these indel differences more likely reflect the use of multiple sequencing platforms utilized in this current study (454 and Illumina data), thereby resulting in the slightly more accurate genome sequence described here. Five SNPs were also identified between genomes, which may be the result of extensive genetic drift between the strains.
